# Evaluation of a novel approach for the measurement of RNA quality

**DOI:** 10.1186/1756-0500-3-89

**Published:** 2010-04-01

**Authors:** Timothy M Wilkes, Alison S Devonshire, Stephen LR Ellison, Carole A Foy

**Affiliations:** 1 LGC, Queens Road, Teddington, Middlesex, TW11 0LY, UK

## Abstract

**Background:**

Microarray data interpretation can be affected by sample RNA integrity. The ScreenTape Degradation Value (SDV) is a novel RNA integrity metric specific to the ScreenTape^® ^platform (Lab901). To characterise the performance of the ScreenTape^® ^platform for RNA analysis and determine the robustness of the SDV metric, a panel of intentionally degraded RNA samples was prepared. These samples were used to evaluate the ScreenTape^® ^platform against an alternative approach for measuring RNA integrity (Agilent Bioanalyzer RIN value). The samples were also subjected to microarray analysis and the resulting data correlated to the RNA integrity metrics.

**Findings:**

Measurement of SDV for a panel of intentionally degraded RNA samples ranged from 0 for intact RNA to 37 for degraded RNA, with corresponding RIN values ranging from 10 to 4 for the same set of samples. SDV and RIN scales both demonstrated comparable discrimination between differently treated samples (RIN 10 to 7, SDV 0 to 15), with the SDV exhibiting better discrimination at higher degradation levels. Increasing SDV values correlated with a decrease in microarray sample labelling efficiency and an increase in numbers of differentially expressed genes.

**Conclusions:**

The ScreenTape^® ^platform is comparable to the Bioanalyzer platform in terms of reproducibility and discrimination between different levels of RNA degradation. The robust nature of the SDV metric qualifies it as an alternative metric for RNA sample quality control, and a useful predictor of downstream microarray performance.

## Background

The use of microarray technology has revolutionised the fields of molecular biology and genetics. However, concerns have been raised over the numerous potential sources of variation that can affect assay consistency and data quality [1,2]. Previous studies have highlighted RNA integrity as one source that has a major effect on microarray data quality [3-5].

To date, no single RNA integrity metric has been adopted universally by the research community. RNA quality is commonly determined by several different techniques, including the ribosomal peak ratio (eukaryotic 28s/18s rRNA peak intensity ratio) [6], RIN [7], 5'/3' transcript signal intensity ratio determined by qRT-PCR or microarray analysis [8] and other quality indices [9].

RNA purity is assessed routinely by measuring the OD260 nm/OD280 nm ratio [6,10,11] of a sample. However, this metric yields no information about RNA integrity. Molecular biologists have therefore relied on the technique of gel electrophoresis, which provides a reproducible separation of ribosomal RNA (rRNA) molecules to derive overall sample RNA integrity. Currently, such conventional methods are being replaced by microfluidic-based platforms, such as that developed by Agilent Technologies (2100 Bioanalyzer).

More recently, Lab901 have developed a novel electrophoretic ScreenTape^® ^platform that employs precast multilane gels and microfluidics enabling semi-automated operation, simplifying sample handling and reducing assay times. In this study we have compared the performance characteristics of the ScreenTape^® ^R6K platform (Lab901) and corresponding RNA quality metric, SDV [9] with the 2100 Bioanalyzer and associated quality metric, RIN [7].

We report here on the broad correlation observed for these quality metric values and associated microarray data.

## Methods

### RNA preparation

HepG2 cells (passage = 84) were grown to confluence in T175 vented culture flasks, using Eagles Minimum Essential Medium (EMEM, ATCC) plus 10% Foetal Calf Serum (FCS, Invitrogen) in a humid 37°C incubator supplemented with 5% CO_2_. Cells were then exposed for 24 h to EMEM exposure media supplemented with 0.5% (v/v) DMSO vehicle (Sigma Aldrich) and 4 mM ACAP (Paracetamol, Sigma Aldrich). Following treatment, the cells were washed with an excess of 1 × Phosphate Buffered Saline (Gibco) before being lysed *in situ *by the application of ice cold TRIzol^® ^LS Reagent (Invitrogen). Total RNA was then isolated according to manufacturer's instructions (Invitrogen). RNA quantity was determined using a NanoDrop 1000 spectrophotometer.

### Instrumentation

For comparative analysis of RNA integrity, the TapeStation^® ^(Lab901) was used in conjunction with ScreenTape^® ^R6K, and the 2100 Bioanalyzer (Agilent Technologies) with the RNA 6000 series II Nano LabChip analysis kit. Total RNA samples were prepared for analysis according to manufacturer's recommendations. Results were compared between the platforms for six levels of RNA sample integrity and at a single RNA concentration of 25 ng/μl.

### RNA degradation

RNA from the treated HepG2 cells was diluted to a concentration of 1 μg/μl with nuclease-free water (Ambion) and aliquoted into volumes of 50 μl in 0.2 ml thin wall PCR tubes. The tubes were placed in the block of an MJ Research PTC-200 DNA Engine Thermal Cycler PCR machine which was then heated to 90°C. Tubes were removed in batches of three at 3-minute intervals. The RNA was then diluted to 25 ng/μl with nuclease-free water, and RNA integrity for each sample determined on both platforms.

### Microarray experimental design

A single-colour labelling approach was adopted for the microarray hybridisation scheme. Duplicate biological samples of those employed for the Lab901/Bioanalyser platform evaluation were used, with two technical replicates (arrays) for each sample. Samples were hybridized to Agilent *Homo sapiens *4 × 44K whole genome gene expression arrays according to manufacturer's instructions. The hybridised arrays were scanned using an Agilent G2505B Scanner and expression data extracted using Feature Extraction software, version 10.5 (Agilent Technologies). Data was exported to the Genespring GX (Agilent) software package, normalised to the 75th percentile of the data set and base-lined to the median signal intensity of all chips. The data were quality controlled by filtering on flags (features present and marginal), before the number of differentially expressed genes (DEG) were determined using the combination of an analysis of variance (ANOVA) and relative fold change (FC = > 1.5).

### Statistical treatment

All statistical analysis used R version 2.9.2 [12].

## Results and discussion

### RNA Integrity Measurements

**Figure 1:**
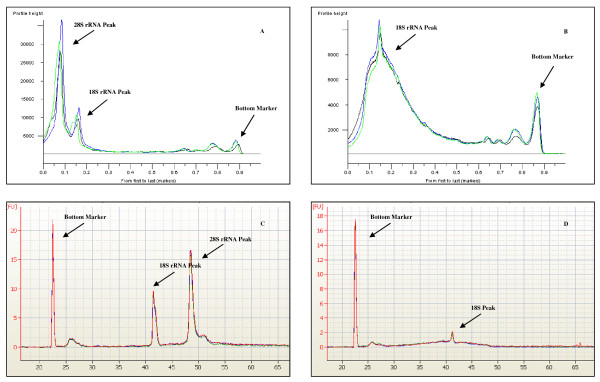
**SDV and RIN chromatograms**. Graphical overlay of SDV chromatograms for the analysis of intact (A), and degraded (B) RNA. RIN chromatograms for intact (C) and degraded (D) RNA are shown for comparison.

RNA integrity was assessed for RNA samples with increasing levels of degradation using the Lab901 ScreenTape^® ^system, which generates SDV values, and the Agilent Bioanalyzer, which generates RIN values. Replicate samples were run on three separate chips or tapes, depending on the platform, and across triplicate lanes per chip or tape. An overlay of chromatograms generated by the ScreenTape^® ^and Bioanalyzer systems for intact and degraded RNA is presented in Figure 1.

### Comparison with RNA Integrity Number (RIN)

**Figure 2:**
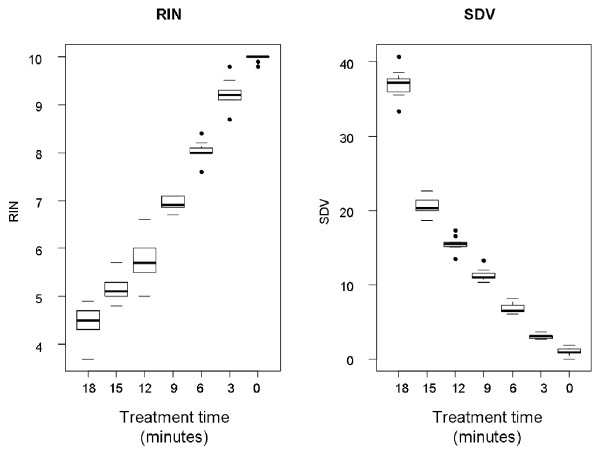
**Relationship between SDV and RIN**. Comparison of the SDV and RIN integrity metrics for a panel of seven RNA samples. "Treatment" denotes the duration of thermal degradation treatment used. Each group includes nine observations, except for treatment four which corresponds to a 12-minute incubation at 90°C and, which includes only seven observations. The thicker horizontal line shows the median, boxes show the upper and lower quartiles and whiskers extend to the most distant data point within 1.5 times the interquartile range of the relevant quartile. Values beyond this are shown as individual data points.

RIN is an incremental scale which spans from 0 to 10, with increasing RNA integrity correlating with increasing RIN value. In contrast, SDV is an unconstrained metric which employs a scale of measurement spanning from 0 to infinity with higher values corresponding to increased degradation. A direct comparison of SDV to RIN was carried out to evaluate the performance of the SDV metric. Preliminary inspection of the data for both platforms (Figure 2) illustrates several important features:

(i) Both RIN and SDV show good discrimination between samples in general.

(ii) SDV appears to show generally better within-group precision.

(iii) There is evidence to suggest that RIN and SDV do not share a linear relationship.

(iv) The within-group dispersion does not appear to be constant across treatment groups for either of the metrics.

### Performance comparison

The assessment of relative platform performance was determined by the use of three different statistical indicators, namely rank order correlation, intraclass correlation and classification performance

#### i) Rank order correlation

Spearman (*ρ*) and Kendall (τ) rank correlations between response and RNA integrity level are listed in Table 1. Both correlation measures indicate very good performance, with the SDV performance appearing marginally better. However, based upon bootstrapping studies (5000 resamples with 95% BCa confidence interval [13] calculated for the difference in magnitude), the difference in rank correlation is not significant at the 95% level of confidence.

**Table 1:** Rank correlations (observed vs. treatment level)

	Spearman *ρ*	Kendall *τ*
RIN	0.984	0.927
SDV	-0.990	-0.934

Spearman (*ρ*) and Kendall (τ) rank correlations between observed RNA integrity level and degradation treatment time.

#### ii) Intraclass correlation

Table 2 lists the calculated intraclass correlation coefficients (ICC) for each metric with respective calculated confidence intervals.

**Table 2:** Intraclass correlation coefficients

	ICC*	95% Confidence interval**
RIN	0.981	(0.949, 0.997)
SDV	0.992	(0.979, 0.998)

* Intraclass correlation coefficient ICC(1) from reference [15]

** Calculated as in reference [15]

Calculated intraclass correlation coefficients with associated calculated confidence intervals.

The ICC for both metrics are high, which is indicative of good performance. The observed ICC for SDV is marginally higher but the confidence intervals show that the difference is not significant at the 95% level of confidence.

#### iii) Classification performance

Four supervised classification methods (rank ordering, Gaussian, K-nearest neighbour (KNN) and linear discrimination analysis (LDA)) were applied to the data sets and the resulting groupings compared with the expected groupings. The significance for differences in the proportion of misclassifications between the RIN and SDV metrics were then tested for each of the classification methods employed. Table 3 summarises these results in terms of the number of misclassifications observed and associated *p*-values.

**Table 3:** Misclassification rates

Classification method	RIN	SDV	*p*-value
Sample ranking	8/61	0/63	0.009
Gaussian classifier	7/61	1/63	0.061
KNN	7/61	0/63	0.017
LDA	8/61	2/63	0.089

Table shows the number of misclassified observations and the total number of observations (two observations were missing from the 9-minute treatment set). All the observed misclassifications were found to be assigned to adjacent treatment groups. Note that for KNN, the misclassification rate varies from run to run due to random tie-breaking (values from 6 to 8 were observed for the 9-minute treatment); the value of 7 given here for KNN was the modal value for 100 KNN reruns.

It can be seen that the relative performance of the two RNA degradation metrics is consistent across all four classification methods. However, more misclassifications were observed when using the RIN metric. In addition, the difference in misclassification was found to be significant at the 95% level for two of the four classification methods. This would indicate that a significant difference in performance exists between the SDV and RIN metrics.

### Microarray analyses

#### RNA integrity and labelling efficiency

**Figure 3:**
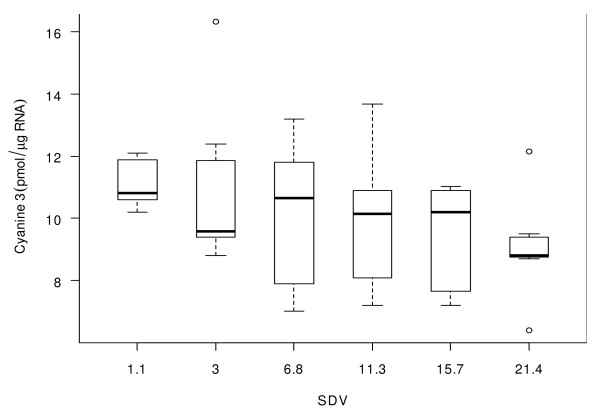
**Relationship of labelling efficiency to sample RNA integrity**. The impact of RNA integrity on microarray sample labelling as evaluated by measurement of Target Specific Activity (Cyanine 3 (pmol/μg RNA)) for an identical mass of input RNA at each level of integrity.

Gene expression studies are potentially sensitive to the effect of RNA integrity. To determine the broad impact of RNA integrity on microarray data, and to determine the value of the SDV metric in highlighting potential problems, microarray labelling data was generated for the degraded RNA samples used in this study. The procedure recommended by Agilent for the amplification and labelling of mRNA samples employs a 3' mediated Eberwine amplification protocol [14] that introduces a directional bias into the pool of synthesised cRNA. Progressive degradation of the RNA template will reduce the population fragment size and is expected to lead to a reduced labelling efficiency. Plotting of the labelling data demonstrates that reduced RNA integrity leads to reduced sample labelling (Cyanine 3 (pmol/μg RNA)) (Figure 3).

In terms of microarray assay performance, reduced sample labelling efficiency may have an impact on the robustness of microarray measurement and data reliability. RNA integrity measurement, whether by SDV or RIN, may provide a valuable tool for early prediction of RNA labelling efficiency and ultimately of overall microarray performance.

#### Microarray data

Assay sensitivity (the rate of detection of true positives) and specificity (the rate of detection of true negatives) are important performance indicators for microarray experiments. To gain insight into the broad impact of RNA integrity, as determined by SDV, a preliminary gene expression experiment was performed using the samples from this study. For each data set generated using different levels of RNA integrity, the number of differentially expressed genes (DEGs) was determined by comparing the paracetamol-treated HepG2 cells to intact RNA extracted from HepG2 cells treated with vehicle control only (DMSO). To assign a measure of assay specificity to the data, a value for the degree of additional gene discovery ("Additional Discovery Rate", ADR) at each level of RNA integrity was derived by subtracting the median number of Differentially Expressed Genes (DEGs) for the technical replicates of the intact sample from the median number of DEGs determined for each of the degraded samples (Table 4).

**Table 4:** The number of differentially expressed genes and associated additional gene discovery rate at each level of RNA integrity

Treatment Time (min)	Measured RIN	Measured SDV	Median number of genes detected*	Additional Discovery Rate
0	10	1.3	5948	0

3	9.2	2.9	7509	1561

6	7.8	6.5	8896	2948

9	7	11.2	10129	4181

12	5.5	15.4	10621	4673

* Genes demonstrating a statistical difference from DMSO sample (P ≤ 0.05) and with a fold change ≥ 1.5

Differential expression was defined as those genes which demonstrated a statistically significant difference of expression (p ≤ 0.05) while employing a Benjamini and Hochberg [15] false discovery rate correction factor, and a fold change ≥ 1.5 compared to the control. The additional gene discovery rate was determined by subtracting the median number of genes determined for three technical replicates of an intact RNA sample away from the median number of genes derived from three technical replicates at each level of degraded RNA sample.

It has been reported previously that gene expression profiling using Affymetrix GeneChip arrays is relatively tolerant to moderate RNA degradation as well as to 5' truncation occurring as a consequence of successive rounds of *in vitro *transcription [16]. However, with progressively decreasing RNA integrity, a substantial increase in the rate of detection of additional positives is reported here, particularly with RIN values < 7 [3]. Comparing RIN or SDV with the ADR reveals a progressive increase in the ADR with decreasing RNA integrity. The exact nature of this increase is uncertain at this stage, but these findings indicate that a shift in assay specificity is occurring as a consequence of reduced RNA integrity, which can be accurately measured and predicted with SDV and RIN.

## Conclusions

The measurement of gene expression is based on the assumption that an analysed RNA sample accurately represents the population of transcripts present *in vivo *[17]. Many transcripts demonstrate stability differences of several orders of magnitude *in vivo *[18], raising the possibility that partial sample degradation could cause variable bias in transcript quantification. The adoption of a suitable RNA quality metric with the capacity to accurately determine RNA integrity is therefore an essential prerequisite for robust data generation in any expression profiling experiment.

In this paper we have compared both the performance of the novel Lab901 ScreenTape^® ^platform with that of the Agilent 2100 Bioanalyzer and also compared the SDV metric with the RIN metric for the determination of RNA integrity when applied to microarray data analysis.

Both metrics performed well when using the samples employed in this study, with the data highlighting the difficulty associated with unambiguously assigning samples to a definitive integrity level when they have ether a RIN value ≤ 6 or an SDV value ≥ 15.

In conclusion, the ScreenTape^® ^system was demonstrated to be a reliable and robust means of determining RNA integrity with SDV estimates, correlating well with the RIN values generated by the Agilent Bioanalyzer platform and with a better classification performance in this study. In addition, the RIN and SDV metrics both performed well in terms of distinguishing different levels of RNA degradation treatment. For microarray data, Rank correlations with treatment and intraclass correlations are high. Classification methods show that the majority of observations were classified into appropriate treatment groups by both RIN and SDV, with the slightly better classification performance of the SDV metric being significant at the P ≤ 0.05 level for two out of the four classification methods used.

The ScreenTape platform and SDV therefore offer an alternative to currently available systems for RNA integrity analysis and provide a performance comparable to that of the Bioanalyzer 2100. The rapid assay time and medium through put capacity may favour its use in laboratories which process large numbers of samples. Future studies with this platform could enable the development of an SDV based RNA quality threshold to be established for use with downstream applications.

## Competing interests

The authors declare that they have no competing interests.

## Authors' contributions

TW participated in the design of the study, carried out the platform evaluations and drafted the manuscript, AD carried out the RNA degradation and SE performed the statistical analyses. CF conceived of the study and participated in its design and coordination and helped to draft the manuscript. All authors read and approved the final manuscript.
